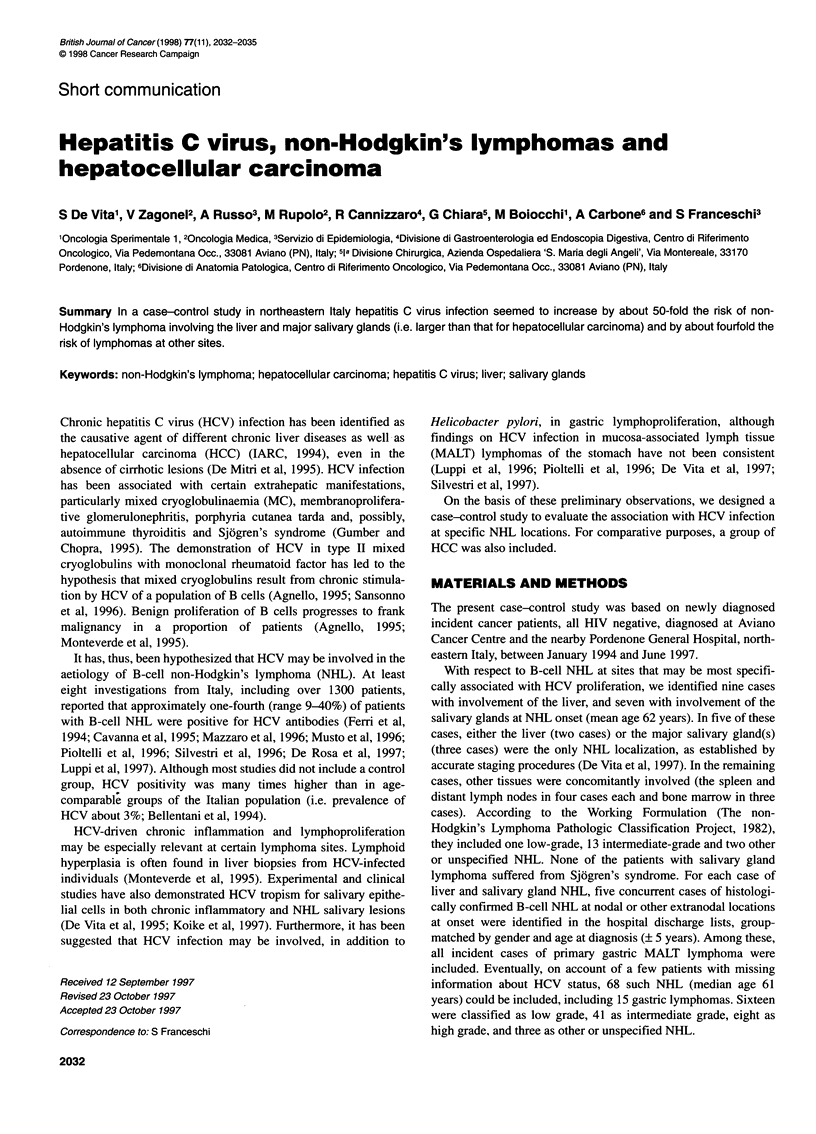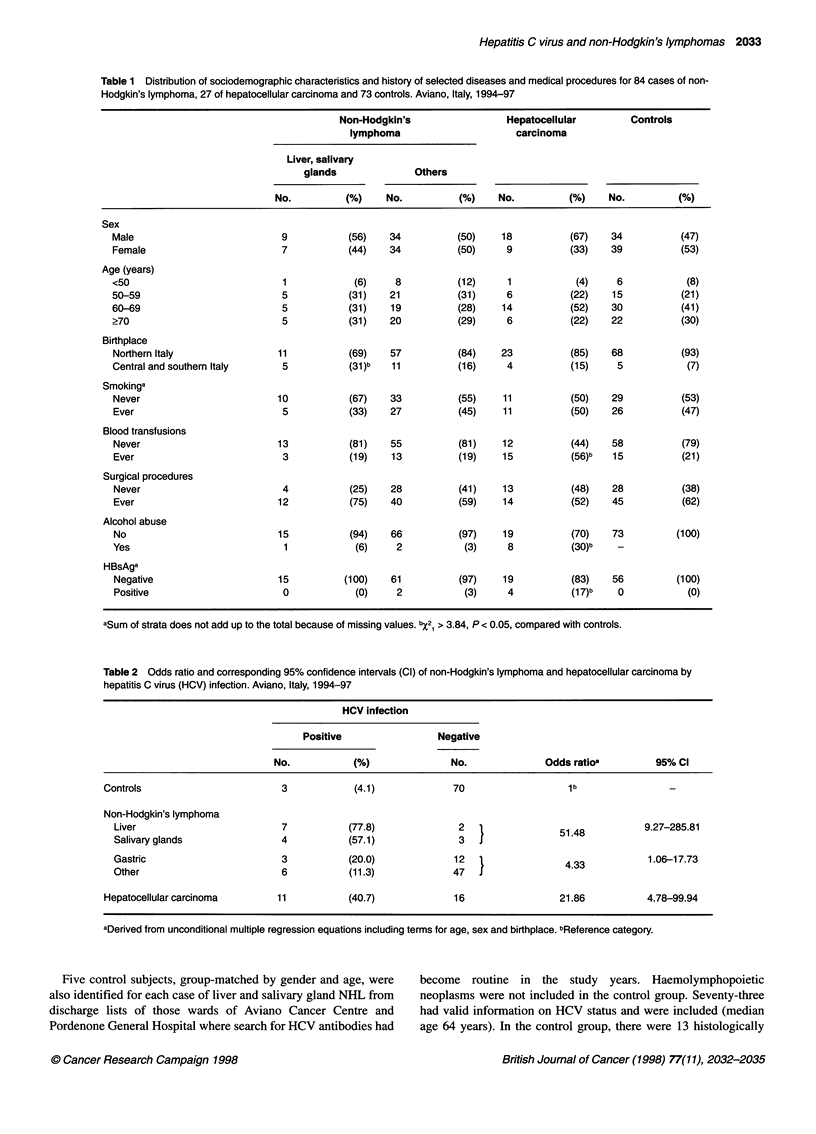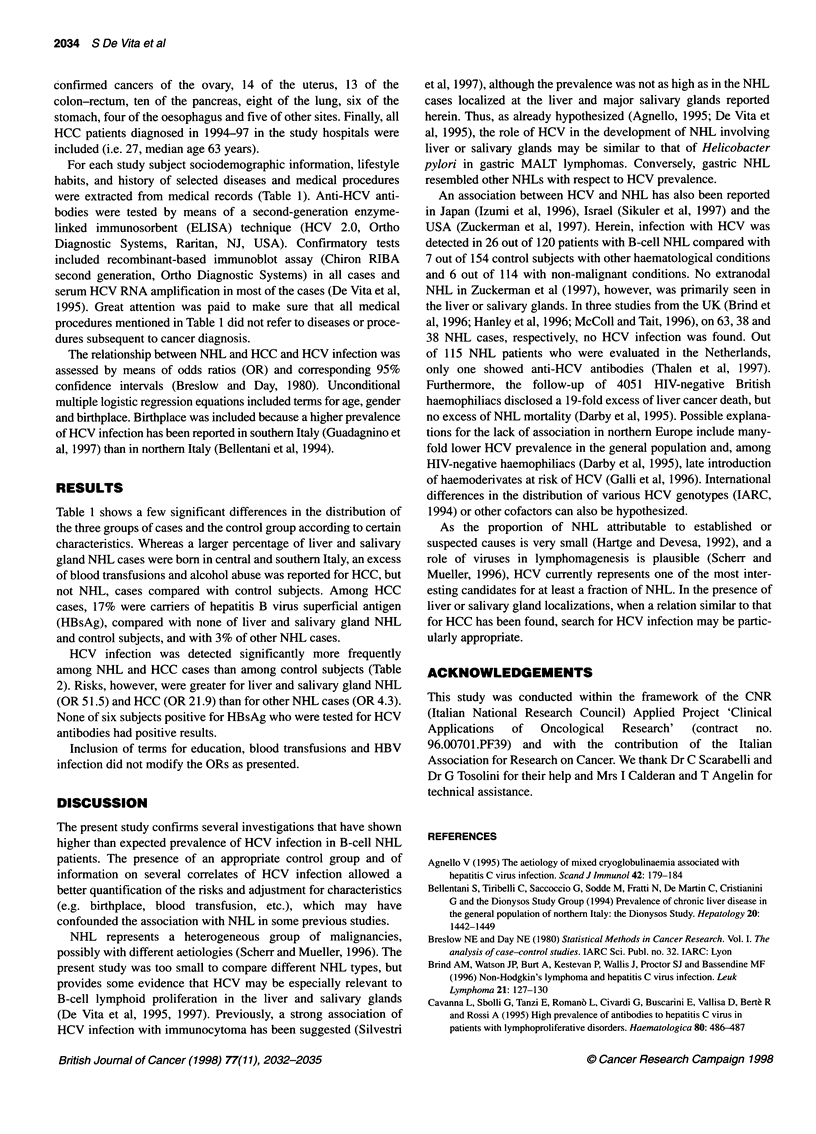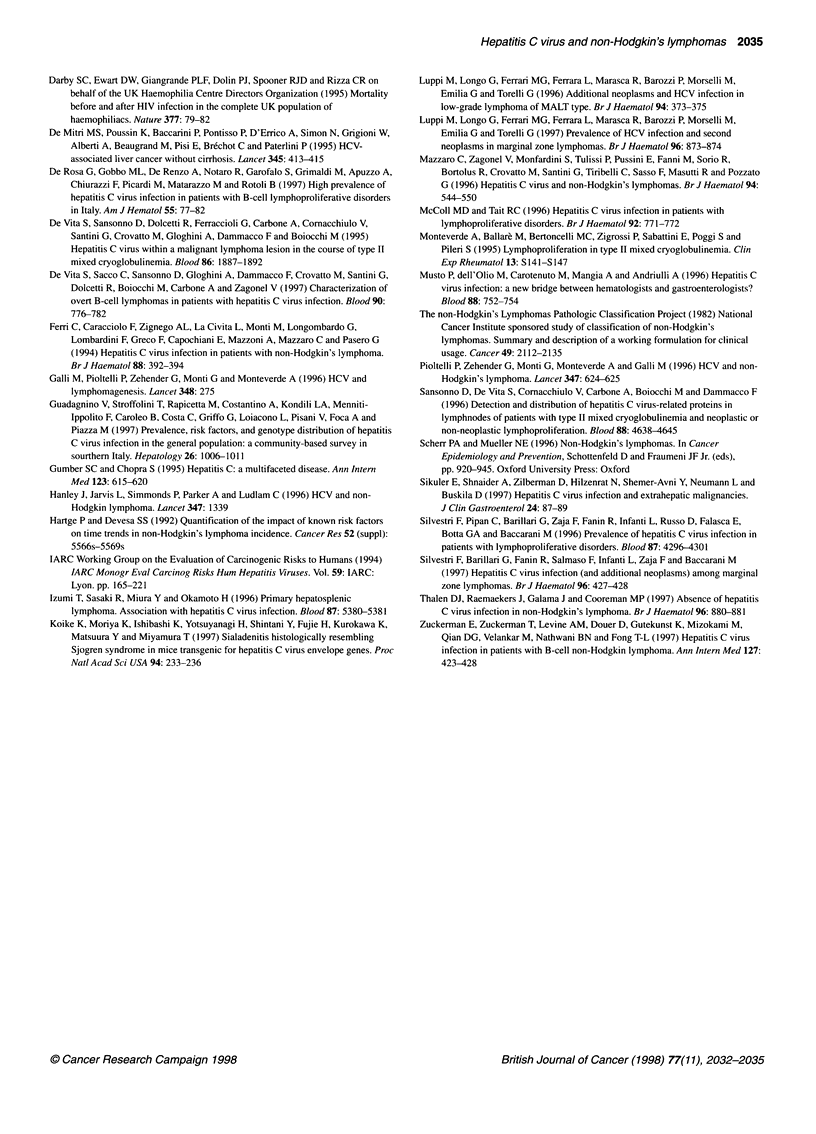# Hepatitis C virus, non-Hodgkin's lymphomas and hepatocellular carcinoma

**DOI:** 10.1038/bjc.1998.338

**Published:** 1998-06

**Authors:** S De Vita, V Zagonel, A Russo, M Rupolo, R Cannizzaro, G Chiara, M Boiocchi, A Carbone, S Franceschi

**Keywords:** non-Hodgkin's lymphoma, hepatocellular carcinoma, hepatitis C virus, liver, salivary glands

## Abstract

In a case-control study in northeastern Italy hepatitis C virus infection seemed to increase by about 50-fold the risk of non-Hodgkin's lymphoma involving the liver and major salivary glands (i.e. larger than that for hepatocellular carcinoma) and by about fourfold the risk of lymphomas at other sites.


*British Journal of Cancer* (1998) **77**(11), 2032–2035
© 1998 Cancer Research Campaign

Short communication

### Hepatitis C virus, non-Hodgkin's lymphomas and hepatocellular carcinoma

**S De Vita[1], V Zagonel[2], A Russo[3], M Rupolo[2], R Cannizzaro[4], G Chiara[5], M Boiocchi[1], A Carbone[6] and S Franceschi[3]**

[1]Oncologia Sperimentale 1, [2]Oncologia Medica, [3]Servizio di Epidemiologia, [4]Divisione di Gastroenterologia ed Endoscopia Digestiva, Centro di Riferimento Oncologico, Via Pedemontana Occ., 33081 Aviano (PN), Italy; [5]Iª Divisione Chirurgica, Azienda Ospedaliera 'S. Maria degli Angeli', Via Montereale, 33170 Pordenone, Italy; [6]Divisione di Anatomia Patologica, Centro di Riferimento Oncologico, Via Pedemontana Occ., 33081 Aviano (PN), Italy

**Summary** In a case–control study in northeastern Italy hepatitis C virus infection seemed to increase by about 50-fold the risk of non-Hodgkin's lymphoma involving the liver and major salivary glands (i.e. larger than that for hepatocellular carcinoma) and by about fourfold the risk of lymphomas at other sites.

**Keywords:** non-Hodgkin's lymphoma; hepatocellular carcinoma; hepatitis C virus; liver; salivary glands

Chronic hepatitis C virus (HCV) infection has been identified as the causative agent of different chronic liver diseases as well as hepatocellular carcinoma (HCC) (IARC, 1994), even in the absence of cirrhotic lesions (De Mitri et al, 1995). HCV infection has been associated with certain extrahepatic manifestations, particularly mixed cryoglobulinaemia (MC), membranoproliferative glomerulonephritis, porphyria cutanea tarda and, possibly, autoimmune thyroiditis and Sjögren's syndrome (Gumber and Chopra, 1995). The demonstration of HCV in type II mixed cryoglobulins with monoclonal rheumatoid factor has led to the hypothesis that mixed cryoglobulins result from chronic stimulation by HCV of a population of B cells (Agnello, 1995; Sansonno et al, 1996). Benign proliferation of B cells progresses to frank malignancy in a proportion of patients (Agnello, 1995; Monteverde et al, 1995).

It has, thus, been hypothesized that HCV may be involved in the aetiology of B-cell non-Hodgkin's lymphoma (NHL). At least eight investigations from Italy, including over 1300 patients, reported that approximately one-fourth (range 9–40%) of patients with B-cell NHL were positive for HCV antibodies (Ferri et al, 1994; Cavanna et al, 1995; Mazzaro et al, 1996; Musto et al, 1996; Pioltelli et al, 1996; Silvestri et al, 1996; De Rosa et al, 1997; Luppi et al, 1997). Although most studies did not include a control group, HCV positivity was many times higher than in age-comparable groups of the Italian population (i.e. prevalence of HCV about 3%; Bellentani et al, 1994).

HCV-driven chronic inflammation and lymphoproliferation may be especially relevant at certain lymphoma sites. Lymphoid hyperplasia is often found in liver biopsies from HCV-infected individuals (Monteverde et al, 1995). Experimental and clinical studies have also demonstrated HCV tropism for salivary epithelial cells in both chronic inflammatory and NHL salivary lesions (De Vita et al, 1995; Koike et al, 1997). Furthermore, it has been suggested that HCV infection may be involved, in addition to *Helicobacter pylori*, in gastric lymphoproliferation, although findings on HCV infection in mucosa-associated lymph tissue (MALT) lymphomas of the stomach have not been consistent (Luppi et al, 1996; Pioltelli et al, 1996; De Vita et al, 1997; Silvestri et al, 1997).

On the basis of these preliminary observations, we designed a case–control study to evaluate the association with HCV infection at specific NHL locations. For comparative purposes, a group of HCC was also included.

### MATERIALS AND METHODS

The present case–control study was based on newly diagnosed incident cancer patients, all HIV negative, diagnosed at Aviano Cancer Centre and the nearby Pordenone General Hospital, northeastern Italy, between January 1994 and June 1997.

With respect to B-cell NHL at sites that may be most specifically associated with HCV proliferation, we identified nine cases with involvement of the liver, and seven with involvement of the salivary glands at NHL onset (mean age 62 years). In five of these cases, either the liver (two cases) or the major salivary gland(s) (three cases) were the only NHL localization, as established by accurate staging procedures (De Vita et al, 1997). In the remaining cases, other tissues were concomitantly involved (the spleen and distant lymph nodes in four cases each and bone marrow in three cases). According to the Working Formulation (The non-Hodgkin's Lymphoma Pathologic Classification Project, 1982), they included one low-grade, 13 intermediate-grade and two other or unspecified NHL. None of the patients with salivary gland lymphoma suffered from Sjögren's syndrome. For each case of liver and salivary gland NHL, five concurrent cases of histologically confirmed B-cell NHL at nodal or other extranodal locations at onset were identified in the hospital discharge lists, group-matched by gender and age at diagnosis (± 5 years). Among these, all incident cases of primary gastric MALT lymphoma were included. Eventually, on account of a few patients with missing information about HCV status, 68 such NHL (median age 61 years) could be included, including 15 gastric lymphomas. Sixteen were classified as low grade, 41 as intermediate grade, eight as high grade, and three as other or unspecified NHL.

*Received 12 September 1997*
*Revised 23 October 1997*
*Accepted 23 October 1997*

*Correspondence to:* S Franceschi

**Table 1** Distribution of sociodemographic characteristics and history of selected diseases and medical procedures for 84 cases of non-Hodgkin's lymphoma, 27 of hepatocellular carcinoma and 73 controls. Aviano, Italy, 1994–97

| | Non-Hodgkin's lymphoma | | | | Hepatocellular carcinoma | | Controls | |
|---|---|---|---|---|---|---|---|---|
| | Liver, salivary glands | | Others | | | | | |
| | No. | (%) | No. | (%) | No. | (%) | No. | (%) |
| Sex | | | | | | | | |
| Male | 9 | (56) | 34 | (50) | 18 | (67) | 34 | (47) |
| Female | 7 | (44) | 34 | (50) | 9 | (33) | 39 | (53) |
| Age (years) | | | | | | | | |
| <50 | 1 | (6) | 8 | (12) | 1 | (4) | 6 | (8) |
| 50–59 | 5 | (31) | 21 | (31) | 6 | (22) | 15 | (21) |
| 60–69 | 5 | (31) | 19 | (28) | 14 | (52) | 30 | (41) |
| ≥70 | 5 | (31) | 20 | (29) | 6 | (22) | 22 | (30) |
| Birthplace | | | | | | | | |
| Northern Italy | 11 | (69) | 57 | (84) | 23 | (85) | 68 | (93) |
| Central and southern Italy | 5 | (31)[b] | 11 | (16) | 4 | (15) | 5 | (7) |
| Smoking[a] | | | | | | | | |
| Never | 10 | (67) | 33 | (55) | 11 | (50) | 29 | (53) |
| Ever | 5 | (33) | 27 | (45) | 11 | (50) | 26 | (47) |
| Blood transfusions | | | | | | | | |
| Never | 13 | (81) | 55 | (81) | 12 | (44) | 58 | (79) |
| Ever | 3 | (19) | 13 | (19) | 15 | (56)[b] | 15 | (21) |
| Surgical procedures | | | | | | | | |
| Never | 4 | (25) | 28 | (41) | 13 | (48) | 28 | (38) |
| Ever | 12 | (75) | 40 | (59) | 14 | (52) | 45 | (62) |
| Alcohol abuse | | | | | | | | |
| No | 15 | (94) | 66 | (97) | 19 | (70) | 73 | (100) |
| Yes | 1 | (6) | 2 | (3) | 8 | (30)[b] | – | |
| HBsAg[a] | | | | | | | | |
| Negative | 15 | (100) | 61 | (97) | 19 | (83) | 56 | (100) |
| Positive | 0 | (0) | 2 | (3) | 4 | (17)[b] | 0 | (0) |

[a]Sum of strata does not add up to the total because of missing values. [b]$\chi^2_1$ > 3.84, $P$ < 0.05, compared with controls.

**Table 2** Odds ratio and corresponding 95% confidence intervals (CI) of non-Hodgkin's lymphoma and hepatocellular carcinoma by hepatitis C virus (HCV) infection. Aviano, Italy, 1994–97

| | HCV infection | | | | |
|---|---|---|---|---|---|
| | Positive | | Negative | | |
| | No. | (%) | No. | Odds ratio[a] | 95% CI |
| Controls | 3 | (4.1) | 70 | 1[b] | – |
| Non-Hodgkin's lymphoma | | | | | |
| Liver | 7 | (77.8) | 2 | 51.48 | 9.27–285.81 |
| Salivary glands | 4 | (57.1) | 3 | | |
| Gastric | 3 | (20.0) | 12 | 4.33 | 1.06–17.73 |
| Other | 6 | (11.3) | 47 | | |
| Hepatocellular carcinoma | 11 | (40.7) | 16 | 21.86 | 4.78–99.94 |

[a]Derived from unconditional multiple regression equations including terms for age, sex and birthplace. [b]Reference category.

Five control subjects, group-matched by gender and age, were also identified for each case of liver and salivary gland NHL from discharge lists of those wards of Aviano Cancer Centre and Pordenone General Hospital where search for HCV antibodies had become routine in the study years. Haemolymphopoietic neoplasms were not included in the control group. Seventy-three had valid information on HCV status and were included (median age 64 years). In the control group, there were 13 histologically confirmed cancers of the ovary, 14 of the uterus, 13 of the colon–rectum, ten of the pancreas, eight of the lung, six of the stomach, four of the oesophagus and five of other sites. Finally, all HCC patients diagnosed in 1994–97 in the study hospitals were included (i.e. 27, median age 63 years).

For each study subject sociodemographic information, lifestyle habits, and history of selected diseases and medical procedures were extracted from medical records (Table 1). Anti-HCV antibodies were tested by means of a second-generation enzyme-linked immunosorbent (ELISA) technique (HCV 2.0, Ortho Diagnostic Systems, Raritan, NJ, USA). Confirmatory tests included recombinant-based immunoblot assay (Chiron RIBA second generation, Ortho Diagnostic Systems) in all cases and serum HCV RNA amplification in most of the cases (De Vita et al, 1995). Great attention was paid to make sure that all medical procedures mentioned in Table 1 did not refer to diseases or procedures subsequent to cancer diagnosis.

The relationship between NHL and HCC and HCV infection was assessed by means of odds ratios (OR) and corresponding 95% confidence intervals (Breslow and Day, 1980). Unconditional multiple logistic regression equations included terms for age, gender and birthplace. Birthplace was included because a higher prevalence of HCV infection has been reported in southern Italy (Guadagnino et al, 1997) than in northern Italy (Bellentani et al, 1994).

### RESULTS

Table 1 shows a few significant differences in the distribution of the three groups of cases and the control group according to certain characteristics. Whereas a larger percentage of liver and salivary gland NHL cases were born in central and southern Italy, an excess of blood transfusions and alcohol abuse was reported for HCC, but not NHL, cases compared with control subjects. Among HCC cases, 17% were carriers of hepatitis B virus superficial antigen (HBsAg), compared with none of liver and salivary gland NHL and control subjects, and with 3% of other NHL cases.

HCV infection was detected significantly more frequently among NHL and HCC cases than among control subjects (Table 2). Risks, however, were greater for liver and salivary gland NHL (OR 51.5) and HCC (OR 21.9) than for other NHL cases (OR 4.3). None of six subjects positive for HBsAg who were tested for HCV antibodies had positive results.

Inclusion of terms for education, blood transfusions and HBV infection did not modify the ORs as presented.

### DISCUSSION

The present study confirms several investigations that have shown higher than expected prevalence of HCV infection in B-cell NHL patients. The presence of an appropriate control group and of information on several correlates of HCV infection allowed a better quantification of the risks and adjustment for characteristics (e.g. birthplace, blood transfusion, etc.), which may have confounded the association with NHL in some previous studies.

NHL represents a heterogeneous group of malignancies, possibly with different aetiologies (Scherr and Mueller, 1996). The present study was too small to compare different NHL types, but provides some evidence that HCV may be especially relevant to B-cell lymphoid proliferation in the liver and salivary glands (De Vita et al, 1995, 1997). Previously, a strong association of HCV infection with immunocytoma has been suggested (Silvestri et al, 1997), although the prevalence was not as high as in the NHL cases localized at the liver and major salivary glands reported herein. Thus, as already hypothesized (Agnello, 1995; De Vita et al, 1995), the role of HCV in the development of NHL involving liver or salivary glands may be similar to that of *Helicobacter pylori* in gastric MALT lymphomas. Conversely, gastric NHL resembled other NHLs with respect to HCV prevalence.

An association between HCV and NHL has also been reported in Japan (Izumi et al, 1996), Israel (Sikuler et al, 1997) and the USA (Zuckerman et al, 1997). Herein, infection with HCV was detected in 26 out of 120 patients with B-cell NHL compared with 7 out of 154 control subjects with other haematological conditions and 6 out of 114 with non-malignant conditions. No extranodal NHL in Zuckerman et al (1997), however, was primarily seen in the liver or salivary glands. In three studies from the UK (Brind et al, 1996; Hanley et al, 1996; McColl and Tait, 1996), on 63, 38 and 38 NHL cases, respectively, no HCV infection was found. Out of 115 NHL patients who were evaluated in the Netherlands, only one showed anti-HCV antibodies (Thalen et al, 1997). Furthermore, the follow-up of 4051 HIV-negative British haemophiliacs disclosed a 19-fold excess of liver cancer death, but no excess of NHL mortality (Darby et al, 1995). Possible explanations for the lack of association in northern Europe include manyfold lower HCV prevalence in the general population and, among HIV-negative haemophiliacs (Darby et al, 1995), late introduction of haemoderivates at risk of HCV (Galli et al, 1996). International differences in the distribution of various HCV genotypes (IARC, 1994) or other cofactors can also be hypothesized.

As the proportion of NHL attributable to established or suspected causes is very small (Hartge and Devesa, 1992), and a role of viruses in lymphomagenesis is plausible (Scherr and Mueller, 1996), HCV currently represents one of the most interesting candidates for at least a fraction of NHL. In the presence of liver or salivary gland localizations, when a relation similar to that for HCC has been found, search for HCV infection may be particularly appropriate.

### ACKNOWLEDGEMENTS

This study was conducted within the framework of the CNR (Italian National Research Council) Applied Project 'Clinical Applications of Oncological Research' (contract no. 96.00701.PF39) and with the contribution of the Italian Association for Research on Cancer. We thank Dr C Scarabelli and Dr G Tosolini for their help and Mrs I Calderan and T Angelin for technical assistance.

### REFERENCES

Agnello V (1995) The aetiology of mixed cryoglobulinaemia associated with hepatitis C virus infection. *Scand J Immunol* **42**: 179–184

Bellentani S, Tiribelli C, Saccoccio G, Sodde M, Fratti N, De Martin C, Cristianini G and the Dionysos Study Group (1994) Prevalence of chronic liver disease in the general population of northern Italy: the Dionysos Study. *Hepatology* **20**: 1442–1449

Breslow NE and Day NE (1980) *Statistical Methods in Cancer Research*. Vol. I. *The analysis of case–control studies*. IARC Sci. Publ. no. 32. IARC: Lyon

Brind AM, Watson JP, Burt A, Kestevan P, Wallis J, Proctor SJ and Bassendine MF (1996) Non-Hodgkin's lymphoma and hepatitis C virus infection. *Leuk Lymphoma* **21**: 127–130

Cavanna L, Sbolli G, Tanzi E, Romanò L, Civardi G, Buscarini E, Vallisa D, Bertè R and Rossi A (1995) High prevalence of antibodies to hepatitis C virus in patients with lymphoproliferative disorders. *Haematologica* **80**: 486–487

© Cancer Research Campaign 1998

Darby SC, Ewart DW, Giangrande PLF, Dolin PJ, Spooner RJD and Rizza CR on behalf of the UK Haemophilia Centre Directors Organization (1995) Mortality before and after HIV infection in the complete UK population of haemophiliacs. *Nature* **377**: 79–82

De Mitri MS, Poussin K, Baccarini P, Pontisso P, D'Errico A, Simon N, Grigioni W, Alberti A, Beaugrand M, Pisi E, Bréchot C and Paterlini P (1995) HCV-associated liver cancer without cirrhosis. *Lancet* **345**: 413–415

De Rosa G, Gobbo ML, De Renzo A, Notaro R, Garofalo S, Grimaldi M, Apuzzo A, Chiurazzi F, Picardi M, Matarazzo M and Rotoli B (1997) High prevalence of hepatitis C virus infection in patients with B-cell lymphoproliferative disorders in Italy. *Am J Hematol* **55**: 77–82

De Vita S, Sansonno D, Dolcetti R, Ferraccioli G, Carbone A, Cornacchiulo V, Santini G, Crovatto M, Gloghini A, Dammacco F and Boiocchi M (1995) Hepatitis C virus within a malignant lymphoma lesion in the course of type II mixed cryoglobulinemia. *Blood* **86**: 1887–1892

De Vita S, Sacco C, Sansonno D, Gloghini A, Dammacco F, Crovatto M, Santini G, Dolcetti R, Boiocchi M, Carbone A and Zagonel V (1997) Characterization of overt B-cell lymphomas in patients with hepatitis C virus infection. *Blood* **90**: 776–782

Ferri C, Caracciolo F, Zignego AL, La Civita L, Monti M, Longombardo G, Lombardini F, Greco F, Capochiani E, Mazzoni A, Mazzaro C and Pasero G (1994) Hepatitis C virus infection in patients with non-Hodgkin's lymphoma. *Br J Haematol* **88**: 392–394

Galli M, Pioltelli P, Zehender G, Monti G and Monteverde A (1996) HCV and lymphomagenesis. *Lancet* **348**: 275

Guadagnino V, Stroffolini T, Rapicetta M, Costantino A, Kondili LA, Menniti-Ippolito F, Caroleo B, Costa C, Griffo G, Loiacono L, Pisani V, Foca A and Piazza M (1997) Prevalence, risk factors, and genotype distribution of hepatitis C virus infection in the general population: a community-based survey in sourthern Italy. *Hepatology* **26**: 1006–1011

Gumber SC and Chopra S (1995) Hepatitis C: a multifaceted disease. *Ann Intern Med* **123**: 615–620

Hanley J, Jarvis L, Simmonds P, Parker A and Ludlam C (1996) HCV and non-Hodgkin lymphoma. *Lancet* **347**: 1339

Hartge P and Devesa SS (1992) Quantification of the impact of known risk factors on time trends in non-Hodgkin's lymphoma incidence. *Cancer Res* **52** (suppl): 5566s–5569s

IARC Working Group on the Evaluation of Carcinogenic Risks to Humans (1994) *IARC Monogr Eval Carcinog Risks Hum Hepatitis Viruses*. Vol. **59**: IARC: Lyon. pp. 165–221

Izumi T, Sasaki R, Miura Y and Okamoto H (1996) Primary hepatosplenic lymphoma. Association with hepatitis C virus infection. *Blood* **87**: 5380–5381

Koike K, Moriya K, Ishibashi K, Yotsuyanagi H, Shintani Y, Fujie H, Kurokawa K, Matsuura Y and Miyamura T (1997) Sialadenitis histologically resembling Sjogren syndrome in mice transgenic for hepatitis C virus envelope genes. *Proc Natl Acad Sci USA* **94**: 233–236

Luppi M, Longo G, Ferrari MG, Ferrara L, Marasca R, Barozzi P, Morselli M, Emilia G and Torelli G (1996) Additional neoplasms and HCV infection in low-grade lymphoma of MALT type. *Br J Haematol* **94**: 373–375

Luppi M, Longo G, Ferrari MG, Ferrara L, Marasca R, Barozzi P, Morselli M, Emilia G and Torelli G (1997) Prevalence of HCV infection and second neoplasms in marginal zone lymphomas. *Br J Haematol* **96**: 873–874

Mazzaro C, Zagonel V, Monfardini S, Tulissi P, Pussini E, Fanni M, Sorio R, Bortolus R, Crovatto M, Santini G, Tiribelli C, Sasso F, Masutti R and Pozzato G (1996) Hepatitis C virus and non-Hodgkin's lymphomas. *Br J Haematol* **94**: 544–550

McColl MD and Tait RC (1996) Hepatitis C virus infection in patients with lymphoproliferative disorders. *Br J Haematol* **92**: 771–772

Monteverde A, Ballarè M, Bertoncelli MC, Zigrossi P, Sabattini E, Poggi S and Pileri S (1995) Lymphoproliferation in type II mixed cryoglobulinemia. *Clin Exp Rheumatol* **13**: S141–S147

Musto P, dell'Olio M, Carotenuto M, Mangia A and Andriulli A (1996) Hepatitis C virus infection: a new bridge between hematologists and gastroenterologists? *Blood* **88**: 752–754

The non-Hodgkin's Lymphomas Pathologic Classification Project (1982) National Cancer Institute sponsored study of classification of non-Hodgkin's lymphomas. Summary and description of a working formulation for clinical usage. *Cancer* **49**: 2112–2135

Pioltelli P, Zehender G, Monti G, Monteverde A and Galli M (1996) HCV and non-Hodgkin's lymphoma. *Lancet* **347**: 624–625

Sansonno D, De Vita S, Cornacchiulo V, Carbone A, Boiocchi M and Dammacco F (1996) Detection and distribution of hepatitis C virus-related proteins in lymphnodes of patients with type II mixed cryoglobulinemia and neoplastic or non-neoplastic lymphoproliferation. *Blood* **88**: 4638–4645

Scherr PA and Mueller NE (1996) Non-Hodgkin's lymphomas. In *Cancer Epidemiology and Prevention*, Schottenfeld D and Fraumeni JF Jr. (eds), pp. 920–945. Oxford University Press: Oxford

Sikuler E, Shnaider A, Zilberman D, Hilzenrat N, Shemer-Avni Y, Neumann L and Buskila D (1997) Hepatitis C virus infection and extrahepatic malignancies. *J Clin Gastroenterol* **24**: 87–89

Silvestri F, Pipan C, Barillari G, Zaja F, Fanin R, Infanti L, Russo D, Falasca E, Botta GA and Baccarani M (1996) Prevalence of hepatitis C virus infection in patients with lymphoproliferative disorders. *Blood* **87**: 4296–4301

Silvestri F, Barillari G, Fanin R, Salmaso F, Infanti L, Zaja F and Baccarani M (1997) Hepatitis C virus infection (and additional neoplasms) among marginal zone lymphomas. *Br J Haematol* **96**: 427–428

Thalen DJ, Raemaekers J, Galama J and Cooreman MP (1997) Absence of hepatitis C virus infection in non-Hodgkin's lymphoma. *Br J Haematol* **96**: 880–881

Zuckerman E, Zuckerman T, Levine AM, Douer D, Gutekunst K, Mizokami M, Qian DG, Velankar M, Nathwani BN and Fong T-L (1997) Hepatitis C virus infection in patients with B-cell non-Hodgkin lymphoma. *Ann Intern Med* **127**: 423–428

*© Cancer Research Campaign 1998*